# Extrapolating from acute to chronic toxicity *in vitro*

**DOI:** 10.1016/j.tiv.2021.105206

**Published:** 2021-10

**Authors:** Peter Macko, Taina Palosaari, Maurice Whelan

**Affiliations:** European Commission, Joint Research Centre (JRC), Ispra, Italy

**Keywords:** *In vitro* toxicity, Acute to chronic extrapolation, Live cell, High content imaging, Chronicity index

## Abstract

Chemical safety assessment requires information on both chronic and acute effects of toxicants. Traditionally, such information has been provided by a set of animal studies conducted over different durations, ranging from a single dose with observation of effects over a few days, to repeat daily dosing and observations made over many months. With the advent of modern mechanistic approaches to toxicology, the role of *in vitro* studies within alternative approaches has never been more prominent. Typical *in vitro* experiments are conducted over short durations with measurements of response at a single time point, with a focus on providing effect and concentration-response information as input to hazard and risk assessment. This limits the usefulness of such data since potential chronic effects that cumulate over time are not usually considered. To address this, an experimental design is presented to characterise the toxicodynamics of a response not only in terms of concentration, but also as a function of time. Generation of concentration-time-effect responses allows both the extrapolation of points of departure from an acute to chronic exposure, and the determination of a chronicity index that provides a quantitative measure of a chemical's potential to cause cumulative effects over time. In addition, the approach provides a means to characterise the dynamics of key event relationships for the development of quantitative adverse outcome pathways.

## Highlights

• Extrapolation of points-of-departure from acute to chronic exposure *in vitro* through time-concentration-response analysis
• Introduction of a chronicity index as a quantitative measure of the cumulative effects a chemical can cause over time
• Experimental approach to characterise the dynamics of key event relationships for quantitative Adverse Outcome Pathways

## 1 Introduction

Typical *in vitro* toxicity methods involve exposing cells to a test item for a period ranging from 6 to 72 h, with an ‘endpoint’ measurement taken when the experiment is terminated. This is essentially an acute toxicity testing scenario that can directly inform acute toxicity assessment of chemicals (Kopp-Schneider et al., 2013), provide mechanistic information useful for hazard identification, and also concentration-effect responses to derive a quantitative Point of Departure (PoD). By applying a suitable means of biokinetic extrapolation, an estimated *in vivo* PoD can be derived from its *in vitro* equivalent (Krause and Goss, 2018; Wetmore, 2015). Although acute toxicity is an important consideration in regulatory toxicology for the protection of human health and the environment, there is considerably more concern about the chronic toxicity of chemicals and their possible contribution to non-communicable diseases such neurodegenerative disorders, impaired fertility and cancer. Thus, for *in vitro* methods to be more broadly useful in risk assessment, they need to be able to identify and characterise the chronic toxicity of chemicals that arises from repeated exposure lasting months, years or even a whole lifetime.

There are several standard chronic and sub-chronic animal toxicity tests that are widely used to characterise chronic toxicity and to satisfy regulatory information requirements (Reichl and Schwenk, 2014). Apart from concerns about the use of animals for toxicity testing, and doubts emerging about the relevance of animal data for informing human health risk assessment, animal tests are undesirable for other reasons too since they require very long exposure times (several months to 2 years), are costly to run, and cannot be feasibly applied to large chemical inventories. Therefore, there is a clear demand for *in vitro* alternatives that have sufficient coverage of relevant toxicological effects and that can be deployed in a cost-effective and high throughput manner. However, if *in vitro* methods require the same long exposure times as conventional chronic toxicity tests using animals then their practical benefits will be severally limited.

Extensive research has been carried out to understand chronic toxicity both for human health and ecological risk assessment. Much of this work has involved the development of models that describe the relationship between the concentration of a toxicant and the time taken to elicit a particular level of an effect in an exposed organism. One well-known example studied by Haber is the relationship between the dose of a toxicant and the time to cause 50% mortality of an exposed population. He observed that for the lethal effects of poisonous gases on animals, the (mathematical) product of the concentration and the exposure time remained constant as either parameter was varied *i.e. Ct = constant*, where *C* is the exposure concentration, *t* denotes time and the *constant* depends on experimental factors. This relationship, known as Haber's rule, is often used in risk assessment contexts as a simple means to extrapolate from one exposure time to another to determine the corresponding dose or concentration at which the same level of effect is expected to happen (European Chemicals Agency, 2016; Gaylor, 2000). Following Haber therefore, doubling the exposure time requires only half the concentration of a toxicant to cause the same level of an effect, and *vice versa*.

**Fig. 1:**
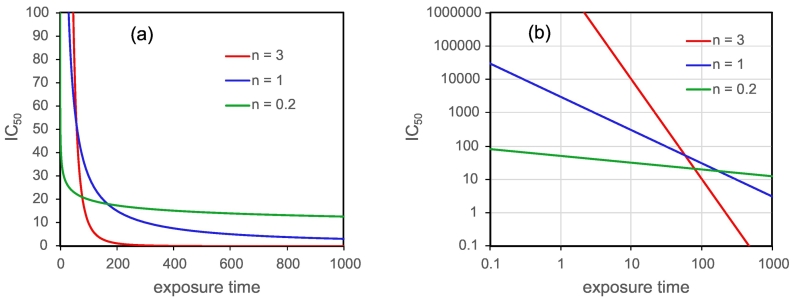
Visualisation of the modified Haber's rule (*C* = *kt*^*-n*^) for three different values of *n*, where the effect-concentration *C* is presented as an IC_50_. Since this is for illustration only, the value of *k* and the units of concentration and time are arbitrary. Left (a) – graph with axes in linear scale shows the ‘hyperbolic’ shape of the relationship. Right (b) – in log-log scale the relationship becomes a straight line, the slope of which is given by the parameter *n*.

As acknowledged by Haber himself (Haber, 1924), and demonstrated by several investigators since (Bliss, 1940; Miller et al., 2000), the original relationship does not always hold true and depends on several factors including which organism or test system is being exposed, the effect or endpoint being measured and the mode-of-action of the toxicant. In fact, a modified Haber's rule where *Ct*^*n*^ *= constant* was found to be more generally applicable (Miller et al., 2000; European Chemicals Agency, 2016; Gaylor, 2000). This relationship can be plotted graphically in two different ways, with either *C* on the x-axis and *t* on y-axis, or *vice-versa*, depending on what one considers the dependant variable. A main interest in the study presented here was to understand how an effect-concentration (*i.e.* the concentration causing a certain level of effect) varies with exposure time *in vitro*, thus we rewrite the modified Haber's rule as *C* = *kt*^*-n*^, where *C* is the dependent variable plotted on the y-axis, *n* ≥ 0, *k* denotes the constant, and *t* is the independent (controlling) variable plotted on the x-axis. As illustrated in Fig. 1a, the relationship between the concentration of a toxicant to elicit a certain level of an effect (*e.g.* IC_50_) and the time needed has a non-linear ‘hyperbolic’ shape. This means for example that in the acute scenario where exposure times are short, the rate-of-change of the effect-concentration with respect to exposure time (*i.e.* the slope of the curve) is greater than in a chronic scenario where exposure times are long. Thus generally speaking, effect-concentrations such as IC_50_, or a PoD such as a Bench Mark Dose (BMD), derived from a concentration- or dose-response experiment for a certain exposure time, would not be expected to change very much when extrapolated within a chronic scenario (*i.e.* from ‘long to longer’ exposure times), whereas in the acute scenario, a derived IC_50_ or PoD may change significantly with small changes in exposure time.

As illustrated in Fig. 1b, the *C* = *kt*^*-n*^ relationship becomes linear when the graph axes are transformed to a log-log scale, with the slope being given by the parameter *n*. The same can be achieved by using the log transformation of the relationship itself *i.e.* log *C* = log *k* – *n* log *t*. This provides a simple means to graphically extrapolate from one set of dose-time conditions to another. Linearity (on the log-log scale) is usually observed across a significant portion of the lifespan of an organism, except for very short times where there might be a lag between exposure and effect or between the effect and being able to detect it, and at very long times when natural mortality starts to play a role. When *n* = 1, we revert to Haber's rule which is often used as a baseline or comparator when analysing concentration-time responses. When testing chemicals which follow Haber's rule, one can expect that in determining a PoD for hazard or risk assessment, increasing the exposure time will result in a proportional decrease in the PoD. In extrapolating therefore from a 90-day (subchronic) to a 2-year (lifetime) rodent study, one would reduce the PoD by a factor of 8, which is close to the default value of 10 often used in regulatory assessments to derive a lifetime reference dose (*e.g.* acceptable daily intake, ADI) from a subchronic study (Gaylor, 2000). When *n* is greater than 1, proportionality is lost and an extrapolated PoD from a shorter to a longer exposure time may well be very much lower than would be expected following Haber's rule. On the other hand, if *n* is less than 1, then the level of toxicity manifest at a certain dose or concentration becomes progressively less dependent on exposure time as *n* gets smaller. In the limit case when *n* approaches 0, the exposure time will have no effect at all and thus the level of toxicity will only be a function of dose or concentration. In pharmacological terms, this is equivalent to a ‘C_max_’ scenario where the magnitude of the response of an organism to a drug is only a function of the maximum (serum) concentration of the drug at the site of action. Other, more complex scenarios are when the value of *n* changes during the period of exposure. For example, an increasing *n* can be indicative of a test system apparently becoming more sensitive to a toxicant as a result of preceding toxicity or due to ageing, as demonstrated for bees exposed to fungicides (Simon-Delso et al., 2018). A decreasing *n* at low concentrations can indicate the existence of a ‘no-effect’ threshold, below which a toxic effect is either not evoked or can even be beneficial. This is the case for some essential nutrients and vitamins (*e.g.* A and D) which are clearly necessary for maintaining health but which can cause adverse effects at high doses and prolonged chronic exposure (Rozman et al., 2010).

Building on this understanding and representation of chronic toxicity derived primarily from experiments involving whole organisms, this study presents the design and demonstration of an *in vitro* approach that provides a means of conducting a short-term *in vitro* assay and extrapolating the results to characterise aspects of chronic toxicity including deriving an *in vitro* chronic PoD.

## 2 Materials and methods

### 2.1 *In vitro* test system

The HepaRG cell line was used as the test system in this study. It was established and patented by the INSERM (National Institute of Health and Medical Research) laboratory at Rennes, France. Undifferentiated HepaRG cells were purchased from Biopredic International (Biopredic, Rennes, France) in cryopreserved vials. After thawing, a cell bank was created to respect the required passage number and frozen again. After subsequent thawing, the cells were seeded into 75 cm^2^ flask at a density of 1 × 10^6^ cells and were cultured in maintenance medium consisting of William's MediumE (Gibco) with 10% FCS (HyClone FetalClone III, HyClone), 2 mM l-glutamine, 1% penicillin/streptomycin, 5 μg/ml bovine insulin, and 50 μM hydrocortisone (all purchased from Sigma). The medium was refreshed every 2 to 3 days. After reaching nearly full confluency, 4 × 10^6^ cells were transferred into 150 cm^2^ flasks and further cultured over 14 days. Their differentiation into hepatocyte-like and biliary-like cells was then initiated by using a differentiation medium consisting of maintenance medium including 0.85% DMSO (purchased from Sigma) for the first day and 1.7% DMSO during following days. After 2 weeks in differentiation medium, the hepatocytes were selectively trypsinized and transferred into black 96-well plates with a transparent flat bottom (Corning 3904), at a seeding density of 5 × 10^4^ cells per well in 100 μl of full HepaRG culture medium without DMSO. The seeded cells were allowed to attach for 72 h before exposure.

### 2.2 Experimental procedure

Cells were treated with 5 different test chemicals: Methylene bis(thiocyanate); Tamoxifen; Cadmium Chloride; Aflatoxin B1 and Rotenone. These chemicals were selected since their toxicological effects and modes-of-action are well known and they are cytotoxic to HepaRG cells at concentrations achievable in the HepaRG cell medium. The cells were treated with 8 different concentrations for a maximum duration of 86 h. The starting concentration and dilution factor were chosen for each chemical in a range-finding experiment with the objective to have a full time-concentration response with good resolution. The dilutions were prepared manually and then transferred to the treated wells. The test plate contained two replicate wells for each chemical at each concentration as well as a number of non-treated (seeded) wells used as negative controls. Immediately after exposure to the test chemicals, the cells were stained with Image-iT® DEAD Green™ (Thermo Fisher Scientific) viability stain. The concentration used was shown in preparatory experiments not to affect cell viability over the longest exposure time (86 h) and this was confirmed by the nominally stable response observed in the negative control wells. The stain is usually used to identify cell membrane damage. It has a high affinity for DNA and forms highly fluorescent and stable dye-nucleic acid complexes while it is non-fluorescent when not bound to DNA. Therefore, the staining of nuclear DNA cannot occur in viable cells due to the impermeability of the cell membrane for the stain, and only dying or dead cells with a compromised cell membrane become fluorescent.

After staining, the 96-well plate was sealed with AeraSeal™ film from Sigma to minimise evaporation, cross-well contamination, and spillage, while allowing uniform air and CO_2_ exchange for all wells. At the end of the experiment the level of the medium in the wells was visually checked and no loss due to evaporation was observed. The well plate was imaged on the High Content Imaging (HCI) Cellomics ArrayScan® VTI instrument (Thermo Fisher Scientific) equipped with a live cell chamber to maintain the cells at 37 °C in humidified atmosphere with 5% of CO_2_ over the total exposure time of 86 h. The HCI instrument, designed for high-capacity automated fluorescence imaging and quantitative analysis of fixed and live cells, was used to acquire and analyse the images. It is built on a fully motorised inverted fluorescence microscope (Zeiss™ Axio Z1 Observer from Carl Zeiss) equipped with an automated stage, objective changer, autofocus system, wheels with different sets of filters and dichroic mirrors for fluorescence channel selection, a highly sensitive CCD camera, and an intense mercury vapour arc light source delivering a broad-spectrum (350 nm–700 nm) illumination through an optical waveguide. The excitation/emission maxima (488/515 nm) of the Image-iT® DEAD Green™ were imaged through the XF93 - FITC filter sets. The instrument was controlled with HCS Studio™ Cell Analysis Software (Thermo Fisher Scientific) which allowed for automated image acquisition, analysis, visualisation, and storage.

The fluorescence images of the stained cell culture were acquired with a 20× objective. All wells on the plate were scanned and in each well four different fields of view were acquired. This was repeated every 30 min over the first 14 h and then every 60 min until 74 h. The final acquisition was made at 86 h. The images were analysed by the Compartmental Analysis v.4 bio-application, which is one of the image analysis tools available in the HCS Studio™ Cell Analysis Software. In each image the bright objects, corresponding to dead or dying cells stained with DEAD Green™, were identified and counted, with the total count serving as a measure for overall cell death. After the experiment, the cells in all wells were fixed and stained with Hoechst 33342 stain. The plate was scanned once more, but now two images for each field of view were recorded, one in the spectral range of Hoechst (XF93 - Hoechst filter set) and one of DEAD Green™. The Hoechst images were analysed and provided the total number of cells in the field of view and the DEAD Green™ image provided the number of dead cells. This provided the basis for data normalisation. For the sake of simplicity and based on previous experience, we neglected the remote possibility of de-differentiation and re-proliferation of HepaRG cells due loss of confluency in treated wells with significant cell death.

## 3 Results

### 3.1 *In vitro* time-concentration response analysis

The viability of the HepaRG cells after exposure to the 5 different toxicants (Methylene bis(thiocyanate), Tamoxifen, Cadmium Chloride, Aflatoxin B1, and Rotenone) was monitored with a non-invasive live-cell fluorescence imaging system as described above, allowing time-concentration responses to be determined for each test chemical.

**Fig. 2:**
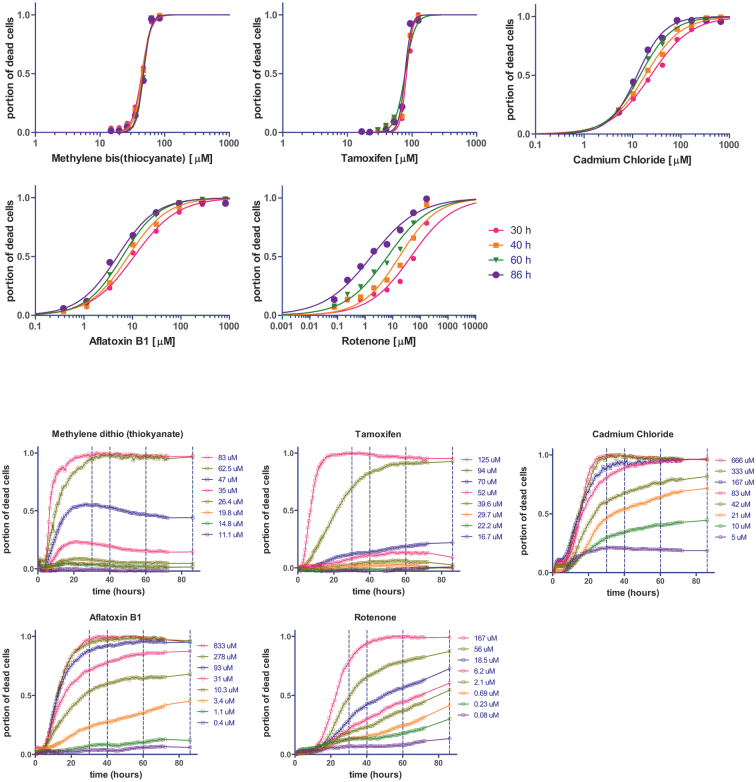
Normalised cell mortality concentration- and time-response curves for HepaRG cell cultures exposed to 5 different chemicals at 8 different concentrations measured over 86 h, top – as a function of the concentration for 4 different exposure times (30 h, 40 h, 60 h, 86 h), bottom – as a function of the exposure time.

The normalised concentration-response curves recorded at four different exposure times (30 h, 40 h, 60 h, 86 h) are shown in Fig. 2 (top) for all 5 chemicals. It can be seen that for Methylene bis(thiocyanate) and Tamoxifen these four responses are practically overlapping, while for the other three chemicals (Cadmium Chloride, Aflatoxin B1, Rotenone) they are progressively shifted to the left (lower concentrations) as the exposure time increases. Fig. 2 (bottom) shows the complete set of time-response curves obtained for each chemical for each concentration tested. The four horizontal dashed lines in Fig. 2 (bottom) mark the time points for which the concentration response curves in Fig. 2 (top) were constructed. The dataset is available at the JRC Data Catalogue (Macko et al., 2021).

**Fig. 3:**
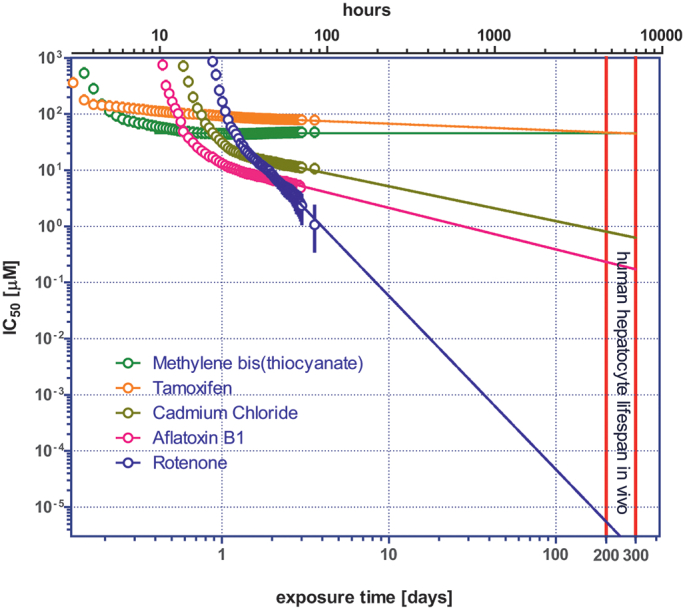
Relationships between the effect-concentration IC_50_ for which 50% mortality of HepaRG cells was observed and the exposure time (open circles). Solid lines show the extrapolation of the IC_50_ to longer exposure times using the modified Haber's rule. The intersections of the extrapolations with the red vertical lines, corresponding to the typical lifespan of hepatocytes in humans (200–300 days), is used to estimate the *in vitro* chronic Point of Departure (cPoD) for each chemical. (For interpretation of the references to colour in this figure legend, the reader is referred to the web version of this article.)

A Hill function was fitted to the normalised data from Fig. 2 (bottom) to generate a concentration-response curve at every experimental exposure time and to derive the half maximal inhibitory concentration, IC_50_, corresponding to 50% mortality of HepaRG cells. The relationships between the effect-concentration IC_50_ for all 5 chemicals and the exposure time are shown in Fig. 3 (open circles). These relationships in the log-log scale are not straight lines in the whole range of the exposure time. The shape of their early parts (up to 10 to 20 h of exposure time, chemical dependent) is affected by several phenomena including the *in vitro* kinetics of the toxicant and the time for the effect to manifest and be detectable. In this study, a dying or dead cell was recorded only at the moment when it became fluorescent thus rendering it detectable by the measurement system. To actually get to this endpoint, a chain of kinetic and biochemical events has to take place. First the tested chemical needs time to be transported from the cell medium to the site of action in the cell; this time is shorter if the chemical acts on the cell membrane as opposed to sites inside the cell (*e.g.* the mitochondria). Having reached the site of action, the chemical triggers an intracellular cascade of events that eventually leads to cell death *via* necrosis or apoptosis. Only when the membrane of a compromised cell becomes permeable can the DEAD Green™ stain enter and find its way to the nucleus to bind to DNA and become fluorescent. The earliest detectable dead cells were observed after around 2.5 h of exposure to the highest concentration of Tamoxifen, and 3.5 h in the case of Methylene bis(thiocyanate). In both cases the cells probably died *via* necrosis. This is supported also by visual observation of the changes of their relative positions from frame to frame (data not shown). It was noted that once the cells became stained and visible, their positions were not seen to change anymore, which suggests quite rapid necrotic death. On the contrary, cells exposed to lower concentrations were seen to slightly change their position from frame to frame, suggesting that they were dying more slowly, *via* apoptosis. It is known that apoptosis can take up to 10 h (Goldstein et al., 2006; Sundquist et al., 2006) indicating a significant delay between initiation (point of no return) and actual terminal cell death.

This process-related kinetics/dynamic ultimately defines the finite response-time of the system which is more evident in the initial phase of the experiment and much less pronounced in later stages of the experiment when the exposure time is longer. Therefore, the initial nonlinear parts of the IC_50_-time curves were not used to either calculate the slope to get *n* or to extrapolate the IC_50_ to longer exposure times, as described below. Another reason why the relationship between exposure time and IC_50_ might not be linear in the early stage of an experiment is the possible saturation of the biomolecular targets with which the toxicant interacts or binds. Once the targets are saturated, increasing the concentration will not make the effect happen earlier. Models to fit these early nonlinear responses based on mechanistic reasoning do exist in the literature however this was not considered important to pursue here.

For the reasons just mentioned, the mean square root method was used to fit the eq. *C = kt*^*-n*^ to only the linear part of the curves (from approximately 30 h to 86 h) to obtain the parameters *n* and *k* for each chemical. The results of the best fits are shown in Fig. 3 as solid lines and the parameters *n* and *k* are provided in Table 1 together with the initial delays *t*_*0*_ to observe the first instance of cell death. The constant *k* has the value of the IC_50_ at one unit of time, which is one day in this case. The fitted lines in Fig. 3 are extrapolated to exposure times corresponding to the typical lifespan of hepatocytes in the human liver which is between 200 and 300 days (Bucher and Malt, 1971), depicted by two vertical red lines. The extrapolation shows how the IC_50_ depends very much on exposure time. The values of the extrapolated IC_50_ at 300 days are also presented in Table 1. Fig. 3 clearly highlights the dramatic difference in IC_50_ values (extrapolated to 300 days) between chemicals whose level of toxicity is primarily dependent on concentration (*n* close to 0) and those for which the level of toxicity they cause increases over time (*n* > 0). As indicated by the data show here, this difference can be extremely high *i.e.* 7 orders of magnitude in the case of Rotenone *versus* Methylene bis(thiocyanate) or Tamoxifen.

**Table 1:** Coefficients *n*, *k,* and initial delay *t*_*0*_ (approximate), together with the extrapolated IC_50_ at 300 days, for the 5 chemicals tested on HepaRG cells.

	*n*	*k* [μM × day^*n*^]	*t*_*0*_ [hours]	IC_50_ at 300 days [μM]
Rotenone	3.1 ± 0.12	70.3	21	1.5 × 10^−6^
Aflatoxin B1	0.74 ± 0.03	11.7	11	0.17
Cadmium Chloride	0.62 ± 0.04	21.5	14	0.63
Tamoxifen	0.12 ± 0.02	88.5	2.5	45
Methylene bis(thiocyanate)	0	46	3.5	46

**Fig. 4:**
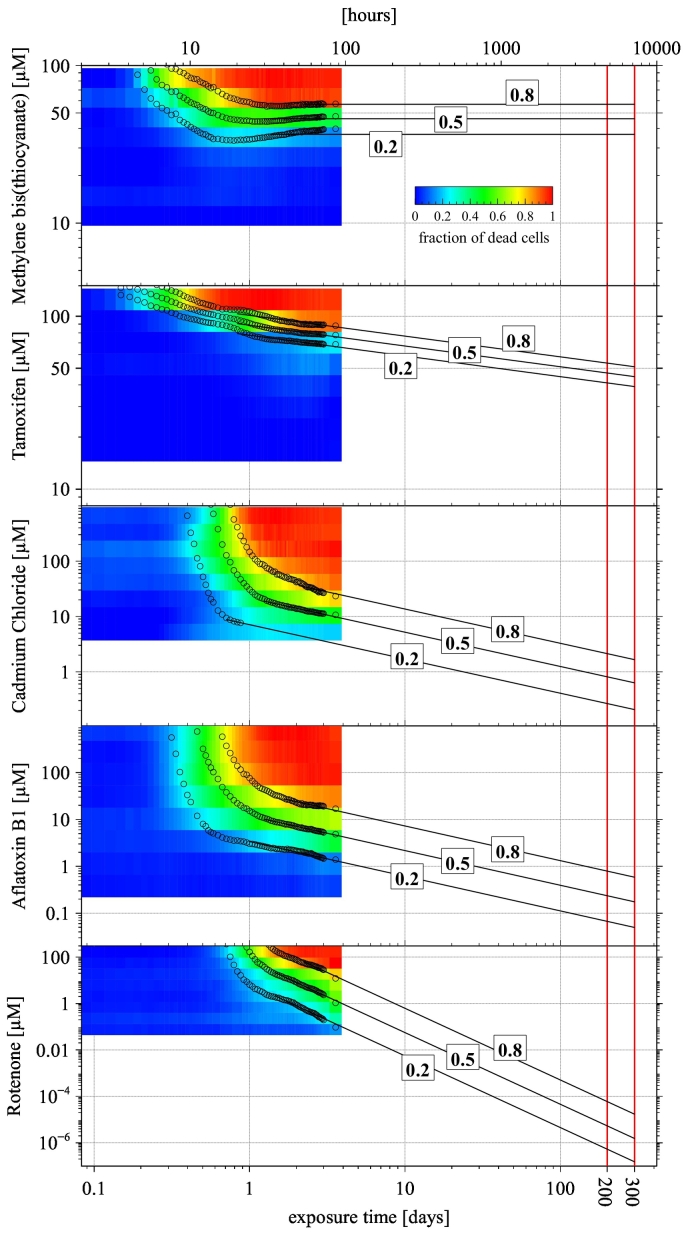
Colour-coded visualisation of the HepaRG cells mortality in concentration *versus* exposure-time domain with black circles showing the points of the isoeffect level (20%, 50%, and 80% cell death). Black lines show their extrapolation up to the typical lifespan of hepatocytes in humans (200–300 days). Note different scales on concentration axes for each chemical.

The set of graphs in Fig. 4 visualise the normalised cell mortality data (from Fig. 2-bottom) in the concentration *versus* exposure-time domain. The colours depict the relative mortality of the cells according to the colour bar (blue – all cells alive, green – half of the cells dead, red – all cells dead). Note that both axes have logarithmic scales and the y-axis (concentration) has different scales for different chemicals. The black circles show the points of isoeffect level (20%, 50%, and 80% of cell death). The points designating the same effect level are fitted with the modified Haber's rule and extrapolated up to the typical lifespan of hepatocytes in humans, for each chemical. This provides a means therefore to derive an *in vitro* chronic PoD (cPoD) from a PoD measured in a short-term assay.

### 3.2 Reconciling *in vitro* response data with *in vivo* observations

Methylene bis(thiocyanate) is a microbicide, fungicide, algaecide and a disinfectant used in a number of contexts including water cooling systems, paint manufacturing, leather processing, and wood protection. It can cause acute liver toxicity (Braun et al., 2006) since it is metabolised in the liver to cyanide and formaldehyde which are both toxic but which can be further biotransformed *via* metabolism to nontoxic compounds. Due to the action of these metabolic toxification and detoxification processes, the toxic effects are not reinforced over time. This is in accordance with the results from the *in vitro* study presented here where only toxicity dependent on chemical concentration was observed. After approximately 20 h of exposure no further cell death was seen to occur.

Tamoxifen is used for the treatment of both early and advanced breast cancer. It can impair fatty acid metabolism in the liver (Ogawa et al., 1998) which can lead to steatosis. It is cytotoxic at higher concentrations and can cause hepatocyte death (Cortes et al., 2019). Tamoxifen is metabolised in the liver by the enzymes CYP2D6, CYP2C9, and CYP3A4 to a toxic metabolite 4-hydroxy-Tamoxifen. However, only rare cases of liver failure have been reported during long-term tamoxifen treatment (Rabaglio et al., 2010), and many of these were also associated with other risk factors. This is consistent with the findings of the *in vitro* study which show that the hepatocyte cell death due to Tamoxifen is dependent on its concentration and as in the case of Methylene bis(thiocyanate), extending the exposure time did not increase the level of toxicity observed.

Cadmium (Cd) is an environmental pollutant and is well known to be toxic to the liver following both acute and chronic exposure (Souza Arroyo et al., 2012). Cd forms strong bonds with proteins and other biomolecules, thereby interfering with biological metal homeostasis and disrupting cellular antioxidant defence. This gives rise to an excess of Reactive Oxygen Species (ROS) causing oxidative stress which ultimately leads to cell death *via* apoptosis. Findings from the *in vitro* study show that prolonged exposure worsens the toxic effects observed, although the value of *n* calculated for Cd was not very high (*n* = 0.62). As the elimination of Cd from the cells is practically negligible (retention time in humans is 10–30 years), it seems that the cells activated protective mechanisms which were able to control the toxicity to a certain extent.

Aflatoxin B1 (AFB1) has been shown to cause a range of acute and chronic toxic effects *in vivo*, including hepatocellular carcinoma. AFB1 is bio-activated in the liver and its primary metabolite covalently binds to RNA, DNA, and other biomolecules resulting in disruption of many cellular processes which can lead to a variety of effects, including genotoxicity and cell death (McKean et al., 2006). Metabolic biotransformation also results in the detoxification of AFB1 (Do and Choi, 2017; Stark, 2005). As observed in the *in vitro* study reported here, the toxicity of AFB1 increases with duration of exposure with a value of *n* = 0.74. This finding makes sense in the context of AFB1 being an agent that undergoes biotransformation that subsequently causes cell death.

Although it is restricted in many regions, Rotenone is used as a pesticide, insecticide, and as a nonselective way of killing fish. It is considered to be moderately acutely toxic to humans causing a range of general symptoms. Rotenone irreversibly inhibits complex I in mitochondria (Efremov and Sazanov, 2011; Heinz et al., 2017) which compromises the production of ATP and results in excess ROS production. High levels of ROS are cytotoxic and can trigger apoptosis, while reduced levels of ATP slow down cellular repair. Since the binding is irreversible, the number of inhibited complex I proteins increases over time as long as new Rotenone molecules are transported to the mitochondria. This results in an appreciable increase in levels of toxicity with continuing exposure, as reflected in this *in vitro* study where a value of *n* *=* *3.1* was determined for Rotenone from its concentration-time-response data. The initial *t*_*0*_ time delay (approximately 20 h) between the start of the experiment and observation of effect (cell death) was the longest of all the chemicals tested. The probable reason is that the cells with inhibited complex I proteins can still survive for a while by using remaining sources of energy in the cytosol before apoptosis is initiated. In addition, as mentioned above, apoptosis as a process in itself has a finite response time. Regarding the *in vivo* effects of Rotenone, there is no evidence to suggest that it specifically acts on the liver. Although it is probably toxic to hepatocytes *in vivo*, the ability of the liver to protect and repair itself likely compensates for the damage caused when the rate of cell death is not excessive. However, cells in other organs are more sensitive to inhibition of energy production, as exemplified in the apparent link between exposure to Rotenone and Parkinson's disease. Impaired or dying neurons cannot be effectively replaced and their excessive death or loss of function in the brain causes irreversible damage to the nervous system (Bal-Price et al., 2018). Therefore, to elucidate the chronic neurotoxicity of Rotenone, one could consider a similar experiment on neurons using extrapolation to estimate a cPoD based on the typical life expectancy of neurons *in vivo*.

## 4 Understanding the mechanistic basis of concentration-time-responses

Toxicological processes can be broken down into sub-processes of toxicokinetics and toxicodynamics. Toxicokinetics is often described as ‘what the body does to the chemical’ through Absorption, Distribution, Metabolism and Excretion (ADME) and it determines the time-course of the relationship between the external concentration of the chemical and its internal concentration at a site of action. Toxicodynamics on the other hand dictates ‘what the chemical does to the body’ and thus describes the actual mechanisms through which a toxicant elicits an effect. Many different conceptual and mathematical explanations of toxicokinetic and toxicodynamic processes and their combinations have been put forward as a basis for modelling chemical toxicity in various organisms (Sánchez-Bayo, 2009). It is instructive to refer to some of these to better understand the results obtained in the *in vitro* study described here.

Possibly the first mechanistic explanation why *n* in the modified Haber's rule can have different values for different toxicants was put forward by Druckrey and Küpfmüller (Druckrey and Küpfmüller, 1949) and elaborated later by others (Rondeau et al., 2014; Tennekes and Sánchez-Bayo, 2013). Although originally proposed for carcinogenic substances the concepts described are more generally applicable. To simplify matters somewhat, it was supposed that the toxicokinetics was steady state and thus only the toxicodynamics determined the slope (*n*) of the relationship between the effect-concentration and the exposure time-to-effect. The toxicodynamics was broken down into two sub-processes: the first, using Adverse Outcome Pathway (AOP) terminology (Villeneuve et al., 2014), is a Molecular Initiating Event (MIE) where the toxicant interacts with a biomolecular target (*e.g.* binding to a receptor). The second is a Key Event (KE), caused by the MIE, which damages the cell. According to Druckrey and Küpfmüller, both the MIE and the KE can be either reversible or irreversible. A reversible MIE would mean, for example, that dissociation of a toxicant-target binding would be possible while a reversible KE would mean that the cell is able to activate repair mechanisms and return to a healthy state. Supposing steady state kinetics (*i.e.* constant external and internal concentrations), there are four possible combinations of these two reversible/irreversible sub-processes. If both the MIE and KE are reversible they will not be cumulative over time and thus the level of effect *E* will be proportional to the internal concentration which is proportional to the external concentration, *E ~ C*, which is equivalent to the modified Haber's rule with *n* *=* *0*. If the MIE is reversible, but the KE is irreversible, the effect will be cumulative and will be proportional to the integral of the concentration over time, *E ~ ∫ C dt*. For constant *C* the integration yields *E ~ Ct*, which is Haber's rule (*n* *=* *1*). The same relationship will be obtained for the opposite combination, an irreversible MIE and a reversible KE. This is equivalent to the pharmacological scenario where an effect is proportional to the ‘area under the curve’ (dose *versus* time). If both, the MIE and the KE are irreversible, the effect will be proportional to the double integral of the concentration over time, *E ~ ∫∫ C d*^*2*^*t*. For constant *C*, the integration yields *E ~ Ct*^*2*^, which is the modified Haber's rule with *n* *=* *2*.

Although very useful to aid understanding of how different toxicodynamic scenarios lead to different characteristics of chronic toxicity, the conceptual model presented by Druckrey and Küpfmüller (Druckrey and Küpfmüller, 1949) is not able to explain a value of *n* greater than 2, although higher values have been experimentally observed, as in the study presented here. A value of *n* > 2 could be explained if one considers a scenario where a toxicodynamic process comprises more than two sequential events. For example, if three distinct events (*i.e.* an MIE and two KEs) are required to cause a particular effect, and if all of these are irreversible, then the magnitude of the effect will be proportional to the triple integral of the concentration over time, *E ~ ∫∫∫ C d*^*3*^*t*. For constant *C*, the integration will thus yield *E ~ Ct*^*3*^. This could be further extended for any integer value of *n*, *n* being the number of sequential irreversible events leading to the effect of interest, with the level of effect being proportional to the n-fold integral of the concentration over time. To recap, the reasoning above assumes that i) the concentration of the toxicant is constant at the site of action, ii) the toxicity pathway can be described by a causally linked sequence of discrete events, and iii) the events are either completely reversible or irreversible. As a consequence, *n* always has an integer value ≥0. However, as with the data presented here, many different experimental setups frequently show *n* to have non-integer values too. This likely happens when the assumptions mentioned above are not fulfilled, for example when the intracellular concentration actually varies over the duration of exposure, the toxicodynamic events are complex in nature (*e.g.* partially reversible, involve feedback/feedforward mechanisms), or the effect observed is actually due to the combined effect of a network of interacting pathways.

Another theoretical derivation of the modified Haber's rule can be found in the work of Breck (Breck, 1988). Inspired by Mancini (Mancini, 1983), he suggested that damage (effect) is proportional to a power of concentration (*C*^*Y*^), and from the differential rate equation for the cumulative damage he derived a relationship which is similar to the modified Haber's rule. Contrary to Druckrey and Küpfmüller, Breck supposed that the effect is linearly proportional to exposure time and thus only the power factor, *y,* of the concentration (*C*^*Y*^) defines the slope. However, Breck (Breck, 1988) didn't provide mechanistic reasoning for his proposition or what the exponent *y* stands for. Actually, the same relationship was proposed several decades before by Ostwald and Dernoschek (Ostwald and Dernoscheck, 1910) and later used by Bliss (Bliss, 1940).

A different approach to derive the modified Haber's rule is shown in the work of Focke et al. (Focke et al., 2017). They proceeded from a general form of the Prout-Tompkins rate equation, also referred to as Sesták-Berggren equation (Brown and Glass, 1999). This rate equation is used in chemistry to describe an autocatalytic reaction (*e.g.* as observed in explosions, thermal decomposition of crystals, or resin-cure reactions), for which at least one of the reaction products acts as a catalyst to accelerate the reaction itself. In toxicological terms, this rate equation can be used to describe the accrual of damage, which is at any time proportional not only to the concentration of the toxicant and the time elapsed, but also to the damage accumulated so far, *i.e.* the damage accrual is a kind of autocatalytic reaction. This hypothesis is particularly interesting to describe the effects of toxicants with *n* > 2.

## 5 Discussion

There are clear demands and opportunities for incorporating New Approach Methodologies (NAMs) into chemical safety assessment across multiple sectors (ECHA, 2016; Knight et al., 2021; Lanzoni et al., 2019; Pistollato et al., 2021). Considerable progress has already been made to demonstrate how NAMs can be used in various contexts including read-across (Escher et al., 2019; Pestana et al., 2021), Integrated Approaches to Testing and Assessment (IATA) (OECD, 2020) and Next Generation Risk Assessment (NGRA) (Baltazar et al., 2020; Berggren et al., 2017). Recently too, it has been shown how *in vitro* bioactivity data generated with high-throughput screening assays can be used to derive a PoD to serve screening-level human health safety assessments (Paul Friedman et al., 2020). Although considerable effort has been invested in developing tools and techniques to derive an *in vivo* (organism level) PoD from its *in vitro* equivalent, such extrapolation usually only takes biokinetic factors into account. Little or no attention is given to extrapolation of toxicodynamic aspects, and in particular, if and how the concentration-effect responses and associated PoDs measured in typical acute *in vitro* setups can be effectively and convincingly extrapolated to chronic *in vivo* scenarios.

Extrapolation of PoDs between different exposure times is frequently used to fill data gaps in regulatory toxicology and risk assessment using simple methods such as Haber's rule. As described above, a considerable amount of investigation has been carried over a hundred years or so to explore dose(exposure)-time-effect relationships in many different species and to understand their mechanistic underpinning. Although the knowledge gained has been put to good practical use in certain situations, it is curious that the concept of ‘chronicity’ is not more prominent in toxicology and hazard assessment. The term is not widely used and has slightly different meanings where it arises (Hayes, 1967; Rozman et al., 2010) but in general it describes the relationship between PoDs derived from acute and chronic studies, expressed quantitatively as a ratio or an index. Lower values indicate a low cumulative rate of effect with increasing exposure time, and *vice versa*. For example, comparing LD50 values derived from acute and 90-day rodent studies shows the significant cumulative effects of warfarin and some chemical biocides, whereas very little cumulative behaviour is observed for caffeine, potassium cyanide and some organic phosphorus compounds (Rozman et al., 2010). Since chronicity can be very high for some substances and vary significantly between substances, it is clearly an important factor to take into consideration when extrapolating to long exposure times (*e.g.* lifetime) and setting safe exposure limits.

In the context of this *in vitro* study, the chronicity index can be defined quantitatively as simply equal to *n*. When *n* is close to or equal to zero, then there is little or no accumulation of effect over time of exposure. As *n* increases, the cumulative rate increases. Determining an *in vitro* chronicity index in this way could provide an important piece of information for hazard and risk assessment. In a screening and prioritisation context for example, chemicals with high chronicity would be of more concern to safety assessors that those with low chronicity. In addition, in cases where extrapolation from shorter to longer exposure times is necessary to define a PoD for safety assessment, then knowing the chronicity might be critical not to either underestimate or overestimate reference values, such as a No-Effect Concentration (NEC) in ecotoxicology or an ADI in human safety assessment. As shown here, if *n* is as large as 2 or 3, and the extrapolation is over a significant timeframe, then the appropriate PoD might well be orders of magnitude less than the PoD measured in the short-term test. One explanation why chronicity is not typically requested for regulatory purposes is that to measure it with suitable precision, several dose-response experiments must be conducted at different time-points which is costly in terms of time, money and use of laboratory animals. However, such barriers do not necessarily exist when using NAMs and thus the determination of a chemical's *in vitro* chronicity index to inform hazard and risk assessment should be feasible and desirable.

The NAM presented in this study provides a science-based approach to extrapolate from acute to chronic toxicity in an *in vitro* testing context to determine two key toxicological parameters, *n* and cPoD. However, there are several sources of uncertainty that should be recognised that go beyond the usual issues associated with *in vitro* methods. These relate both to determining cPoD and *n* and their subsequent extrapolation to a hazard or risk assessment scenario that concerns a particular target species (whole organism). The first is the assumption that after an initial period of transient behaviour, the system effectively reaches a steady-state condition where *n* remains constant. However due to experimental limitations, this steady state could only be observed until the maximum exposure time of 86 h was reached. It may well be the case that even if the experimental conditions could be kept constant over longer periods, the test system itself (HepaRG cell culture in this case) might biologically change over time which would naturally render it more or less sensitive to a particular toxicant, resulting in a change in *n* with exposure time. In fact, this may well be expected since is it not uncommon for a test system to become more sensitive as the level of toxicity it accumulates increases. The opposite is also possible, where a test system actually becomes more resistant to a toxicant over time of exposure. Thus although this steady state has been frequently observed in many whole-organisms (mammals, rodents, fish, insects, bacteria) subject to many different toxicological tests, it is not necessarily the case that such steady states are likely to be a common feature of *in vitro* tests.

The modified Haber's rule used to fit the experimental data generated in this study and to calculate values for *n* and cPoD assumes that the intracellular concentration of the toxicant remains constant over the course of the experiment. This may not have been the case due to the possible effects of *in vitro* biokinetics where, for example, metabolic biotransformation or bioaccumulation can result in concentration changes. There are some attempts described in the literature to mathematically extend the modified Haber's rule to make it applicable to a test where the concentration decays over the exposure time (Bogen and Reiss, 2012; Breck, 1988). However, the increase in complexity of analysis only seems justified if the concentration changes are very significant and the desired level of precision is very high. In the experiments conducted in this study, after roughly 30 h of exposure the concentration-time-effect relationships (log-log) for all 5 chemicals became linear. This indicates that a steady state was likely reached and thus the intracellular concentration of the chemicals was approximately constant during the experiment. Although a change in *n* over time can be practically handled in an assessment context, by taking a worse-case scenario for example, understanding the reasons why *n* varies with exposure time can lead to important insights into experimental conditions and the mechanistic basis to the toxicological response of the test system (Simon-Delso et al., 2018).

The concentration-time-effect responses measured in this study and the chronicity values derived for the chemicals tested are specifically related to the effect or KE measured *i.e.* cell death. Choosing a different effect to measure will give a different concentration-time-effect response and thus a different chronicity index for the same chemical. This is to be expected since each toxicity pathway has its own toxicodynamic behaviour. Therefore, rather than treating chronicity as an intrinsic toxicological property of a chemical, one could instead consider it as a toxicodynamic property of an AOP. Once the chronicity of an AOP is characterised, for example through the development of ‘quantitative’ AOPs (Villeneuve et al., 2014; Wittwehr et al., 2017), any chemical which is shown to activate the associated MIE will ‘inherit’ the chronicity property of that AOP. Thus, two chemicals exhibiting similar biokinetic behaviour and triggering the same AOP are likely to have the same chronicity. Of course, it can also be the case that a chemical simultaneously activates more than one AOP and thus its observed chronicity for the effect being measured will depend on the overall toxicodynamic behaviour of the integrated AOP network.

Exposure time is obviously an important consideration in conventional regulatory testing which relies on a suite of tests covering acute, sub-acute, sub-chronic, and chronic exposures to fully characterise chemical toxicity. Longer duration tests are typically considered of more value in hazard and risk assessment since they are usually seen as being more ‘sensitive’ and thus more protective. In the absence of longer-term studies, extrapolation from shorter to longer exposure times through the application of assessment factors is often the only option since longer-term studies might not be a regulatory information requirement or they might be prohibitive in terms of time, money and use of animals. It is curious therefore that as NAMs have emerged based on *in vitro* methods, the same value or attention has not been given to measuring the toxicodynamics of chemicals with respect to exposure time, with a view to characterising both (sub-)acute and (sub-)chronic toxicity and being able to distinguish and potentially extrapolate between them. Of course several *in vitro* studies have been conducted to investigate the influence of exposure time on outcome (Karri et al., 2018; Gülden et al., 2010; Fotakis and Timbrell, 2006; Abassi et al., 2009; Holmgren et al., 2014; Hsieh et al., 2017; Gu et al., 2018; Proctor et al., 2017; Seibert et al., 2004) but not with a view to providing toxicodynamic information useful in hazard and risk assessment.

One frequently cited motivation for varying exposure time within an *in vitro* study is to maximise the amplitude of the measured effect (signal) or the signal-to-background ratio of the assay employed. This is often conducted as part of method development using positive control chemicals with a view to selecting a single ‘optimal’ exposure time to be used for any chemical subsequently tested. Based on what has been presented here, it is evident that by considering exposure time as a variable along with concentration, single-exposure *in vitro* methods can be adapted to deliver richer information of more value to hazard and risk assessment. Of course, such adaptation to include multiple time-points can lead to technical complications and higher experimental costs. Many *in vitro* assays require cell fixation and cell lysis prior to endpoint measurement, which are obviously destructive procedures by nature. Thus, several cell cultures need to be independently exposed to cover all the required time-points. This can demand significant scaling up of experimental effort and associated costs. However, considering the potential increase in the amount and value of the information produced, the benefits may well outweigh the costs in many cases. An alternative approach to efficiently carry out concentration-time-response studies is to use live-cell monitoring methods where possible. Live-cell assays use non-destructive readouts and thus measurements can be repeated on the same population of treated cells over the course of the experiment. Methods based on live-cell monitoring are also less prone to experimental variability due to differences in cell numbers between wells since the readings at different time points are carried out on the same population of cells.

Depending on the context of use, the assumptions and limitations described here may represent important sources of uncertainty and thus should be taken into account when developing this approach further and applying it for hazard assessment purposes. To summarise, it was supposed that the *in vitro* toxicokinetics reached a steady state for the interval of the response used to determine the chronicity index *n*. Although this assumption seems reasonable for the experiments conducted, knowing more about the actual concentration of the toxicant at the site of action would increase confidence since a changing local concentration would affect the calculation of the chronicity index and the extrapolated cPoD. Another scenario to be mindful of is when the accumulation of effect is very slow, since the duration of the *in vitro* experiment may not be sufficient to identify a situation when a NEC actually exists, in which case the cPoD determined would be lower than reality. Similarly, if no effect is observed, this may not be the case when the same experiment is run with a longer exposure time. The biological stability of the test system over time also needs to be considered to understand if its nominal sensitivity to a particular toxicant varies with exposure time. Other prerequisites for reliable determination of chronicity index and cPoD include obtaining full time-concentration responses (*i.e.* from no effect to full effect) and sufficient measurement resolution, which are not always achievable in some *in vitro* setups.

Finally, in this study the cPoD was calculated through a linear extrapolation of the concentration-time-response curve to an exposure time equivalent to the typical lifespan (200–300 days) of hepatocytes living within human liver. It is questionable if this represents an appropriate time-base for extrapolation since there are obvious biological and micro-environmental differences between primary human hepatocytes *in vivo* and HepaRG cells *in vitro*. These likely translate into respective differences in ‘natural’ lifespan and specific response to a toxicant which lead in turn to uncertainty in deriving a cPoD for safety assessment. This issue therefore requires further consideration.

## 6 Conclusions

This study presents a practical means of measuring the chronicity index of substances *in vitro* and suggests how this important parameter can be used to extrapolate a PoD derived from a short-term experiment to longer exposure times. Moreover, it has been shown how such concentration-time-effect experiments can provide valuable information on the toxicodynamic behaviour of a toxicant not only with respect to concentration, but also with respect to exposure time. This potentially addresses the significant information deficit apparent in current approaches to *in vitro* to *in vivo* extrapolation which usually only take the toxicokinetic component into account. It also encourages developers and end-users to reconsider conventional experimental design, to get more information from their *in vitro* methods by shifting emphasis away from only focusing on acute potency as a primary output, to one which also includes considerations of cumulative effects as a means to better characterise hazard and judge concern. Such an experimental design could also be employed to characterise the dynamics of AOPs and their constituent Key Event Relationships (KERs) as a contribution to the development of ‘quantitative’ AOPs. Providing both a PoD together with an associated chronicity index will increase the relevance and utility of *in vitro* data for hazard and risk assessment and thus enhance the value of NAM derived data in safety decision making.

## Declaration of Competing Interest

The authors declare that they have no known competing financial interests or personal relationships that could have appeared to influence the work reported in this paper.
